# Non-invasive quantification of brain tumor-induced astrogliosis

**DOI:** 10.1186/1471-2202-12-9

**Published:** 2011-01-19

**Authors:** Jisook Lee, Alexandra K Borboa, Andrew Baird, Brian P Eliceiri

**Affiliations:** 1 Department of Surgery, University of California San Diego, San Diego, CA, USA

## Abstract

**Background:**

CNS injury including stroke, infection, and tumor growth lead to astrogliosis, a process that involves upregulation of glial fibrillary acidic protein (GFAP) in astrocytes. However, the kinetics of astrogliosis that is related to these insults (i.e. tumor) is largely unknown.

**Results:**

Using transgenic mice expressing firefly luciferase under the regulation of the GFAP promoter (GFAP-luc), we developed a model system to monitor astrogliosis upon tumor growth in a rapid, non-invasive manner. A biphasic induction of astrogliosis was observed in our xenograft model in which an early phase of activation of GFAP was associated with inflammatory response followed by a secondary, long-term upregulation of GFAP. These animals reveal GFAP activation with kinetics that is in parallel with tumor growth. Furthermore, a strong correlation between astrogliosis and tumor size was observed.

**Conclusions:**

Our results suggest that non-invasive, quantitative bioluminescent imaging using GFAP-luc reporter animal is a useful tool to monitor temporal-spatial kinetics of host-mediated astrogliosis that is associated with glioma and metastatic brain tumor growth.

## Background

The tumor microenvironment is a dynamic niche for tissue remodeling because of its production of tumor cell- and host stromal cell-derived growth factors, cytokines and matrix proteins. Historically, the study of such host-stromal interactions has generally relied on classical histological methods such as immunohistochemistry, in situ hybridization or biochemical techniques such as immunoblotting and enzyme assays. Unfortunately, the analytical power of these techniques is limited by the ability of reagents to distinguish between tumor and host compartments and by the need for terminal harvest of tissues for analysis. For example, in malignant gliomas, tumor cells co-opt the functions of the surrounding brain to support their growth and invasion. However, gliomas fail to completely compromise an otherwise tight blood brain barrier of normal vessels, based on the wide range of drugs and small molecules that fail to cross the BBB and target brain tumors [1].

GFAP expression has been widely used as a marker for astrogliosis and the host response to injury [2-4] and its analysis has generally relied on immunohistochemistry rather than quantification. Recently, however, bioluminescent imaging of GFAP activity using transgenic GFAP-luc mice has been reported to measure astrogliosis in animal models of kainic lesions [5], prion infection [6], ischemic injury [7], and experimental autoimmune encephalomyelitis (EAE)[8], but has not been described in a tumor model. In this study, we used immunodeficient (i.e., Rag2^-/-^) transgenic GFAP-luc mice [5] to grow orthotopic brain tumors and to monitor the co-activation of the GFAP promoter with tumor development. GFAP promoter activation was used as a surrogate marker for host compartment astrogliosis to assess tumor progression. Here we show that these GFAP-luc; Rag2^-/- ^mice injected with malignant glioma cells can be used to monitor and quantify tumor-induced astrogliosis response of the host. Analysis of the serial imaging supports a model in which intracranial tumor injection induces an early GFAP response, which is likely a consequence of the local wounding of the stereotactic injection. This early response resolves and is followed by a secondary astrogliosis response correlating with tumor progression both in terms of kinetics and localization.

## Results and Discussion

### Immunohistochemical analysis of GFAP activity in tumor-bearing brain

**Figure 1:**
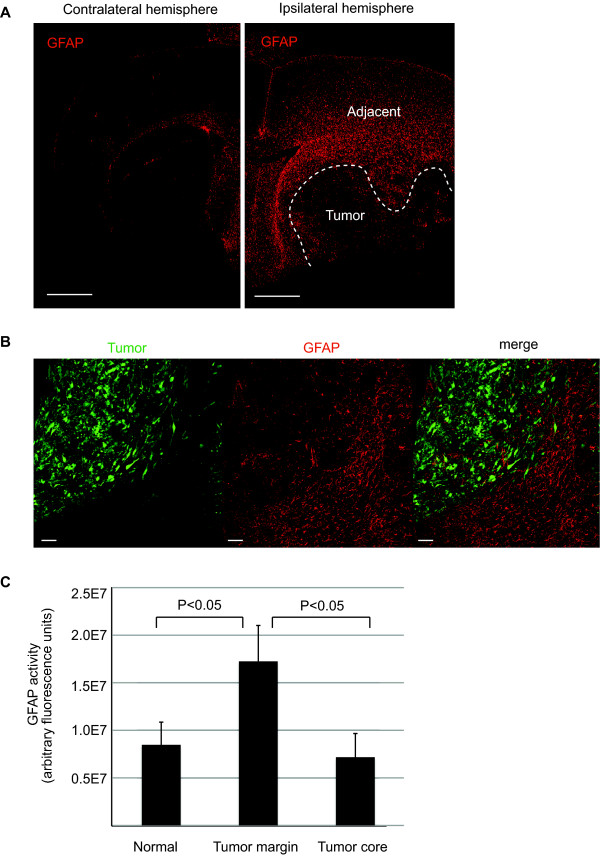
**Using standard immunohistochemical technique to assess GFAP activity in tumor-bearing brain**. (A) DBTRG glioma cells were introduced into mice by stereotactic injection. Following three weeks incubation, brains were harvested and sections were made to measure GFAP activity in tumor bearing vs. contralateral brain by immunohistochemistry. Sections were counterstained with DAPI and tumor area was defined by dense nuclei staining of the tumor cells (dotted line). An example of exposure-matched images of tumor-bearing brain (right) and its contralateral hemisphere (left) is shown. Note the upregulated GFAP activity at the tumor margin (right). Scale bar = 1 mm. (B) Fluorescently labeled DBTRG-Zsgreen glioma cells (green) were introduced into mice by stereotactic injection. Following three weeks incubation, brains were harvested and sections were made to measure GFAP activity (red) by immunohistochemistry. A representative image of upregulated GFAP immunoreactivity at the tumor margin compared to the tumor core is shown. Scale bar = 100 μm. (C) GFAP immunoreactivity in tumor margin (defined by fluorescent activity of the tumor cells), tumor core vs. normal area (contralateral) was quantified by measuring the fluorescent intensity of images in panel (B). Results are shown as an average of three mice. At least three independent areas were quantified from each mouse. P < 0.05, Wilcoxon rank sum test, two-sided. Error bar represents standard deviation.

To determine the effect of orthotopic tumor xenografts on GFAP activity we subjected immunodeficient Rag2^-/- ^mice (i.e. T and B cells defective) [9] to stereotactic injection with DBTRG glioma cells as described in the Methods. These glioma cells have been previously shown to be highly invasive with infiltrative, satellite tumors distant from the primary tumor [10-12]. Both primary and infiltrative tumors induce specific remodeling of the surrounding microenvironment, which has been analyzed by immunohistochemical analyses of cell-type specific markers, for example, GFAP, to detect remodeling and activation of the astrocytes [10]. In agreement with previous observations from our laboratory and others [10,13-16], GFAP activity was highly upregulated at the tumor margin and adjacent tissue forming a glial scar, whereas a decrease in GFAP immunoreactivity was observed in the tumor core (Figure 1). Although GFAP is generally detected in primary glial brain tumors, its expression is often lost in cell lines established from malignant gliomas such as DBTRG cells [17,18]. Thus, scattered GFAP immunoreactivity inside the tumor was likely from infiltrating astrocytes from the host rather than from the human DBTRG tumor cells themselves. Previous observations using a different astrocyte marker, Aldh1L1 (a marker for protoplasmic astrocytes) [19] demonstrate a similar immunostaining pattern with GFAP (i.e. upregulation at the tumor margin, but decrease in the tumor core). However, aquaporin 4 (AQ4, a marker for astrocytic endfeet) immunoreactivity was upregulated in the tumor core and, moreover, contact with endothelial cells was lost inside tumors [10], indicating that distinct subsets of astrocytes react differently upon glioma growth.

While these immunohistochemical techniques are useful for understanding tumor-induced host remodeling at a cellular level, they rely on the availability and validation of species-specific antibodies to distinguish tumor vs host compartment responses. To develop a rapid, quantitative model to better understand the temporal progression of astrocytic gliosis upon tumor growth, we used GFAP-luc reporter animals for a non-invasive detection of tumor-induced astrogliosis.

### Non-invasive imaging analyses of glioma-induced GFAP activation

**Figure 2:**
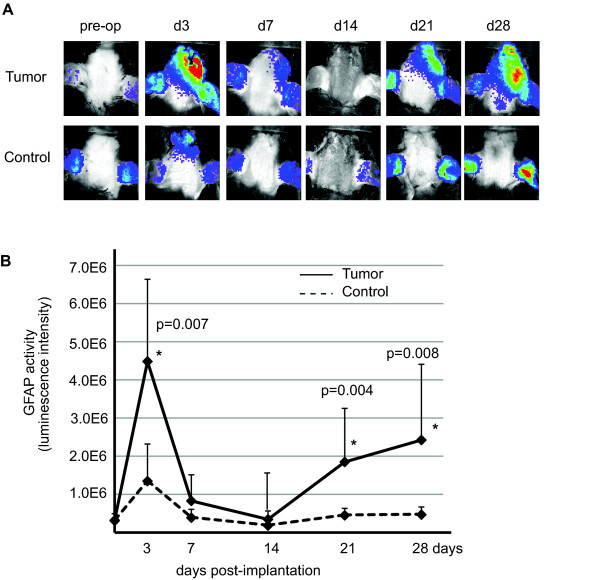
**Non-invasive imaging of glioma-induced GFAP activation**. (A) GFAP-luc mice were injected with RFP-labeled DBTRG human glioma cells (Tumor) or PBS (Control). GFAP activity was monitored for up to four weeks. An exposure-matched, representative non-invasive image of each time point is shown. (B) Quantitative measurement of panel (A). Reactive gliosis was observed three days post-implantation in both tumor and PBS injected animals. Upon tumor growth, GFAP activity increased after 14 day of implantation in animals injected with tumor cells, while only background GFAP activity was observed in animals without tumor. N = 6-7 for each group, Wilcoxon rank sum test, two-sided, * P < 0.01 at day 4, 21 and 28, error bar represents standard deviation.

To determine the effect of orthotopic tumor growth on GFAP promoter-mediated luciferase activity, we backcrossed GFAP-luc mice to an immunodeficient Rag2^-/- ^background. We then injected lentiviral-transduced, DBTRG cells expressing red fluorescent protein (DBTRG-RFP) into the brains of 10 week-old GFAP-luc; Rag2^-/- ^mice by stereotactic injection. Intracranial injections of an equal volume of PBS were used to control for injury induced by the injection itself. As reported earlier [6,20], intracranial injections of PBS alone induced a transient injury response, which was detected at day 3 but diminished by week two (Figure 2). Tumor cells induced a biphasic host astrogliosis response in which tumor cells initially induced GFAP-luc levels that was higher than control followed by a reduction to background by day 7. However, further tumor growth after three and four weeks led to second, larger, sustained and statistically significant five-fold increase of GFAP activity over PBS controls (Figure 2).

### Non-invasive imaging analyses of metastatic brain tumor-induced GFAP activation

**Figure 3:**
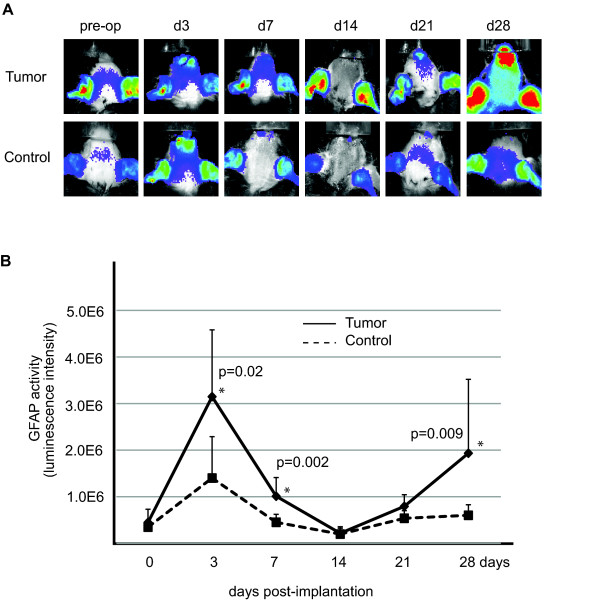
**Non-invasive imaging of metastatic brain tumor-induced GFAP activation**. (A) GFAP-luc mice were injected with MDA-MB-231 breast tumor cells (Tumor) or PBS (Control). GFAP activity was monitored for up to four weeks. An exposure-matched, representative non-invasive image of each time point is shown. (B) Quantitative measurement of panel (A). Reactive gliosis was observed three days post-implantation in both tumor and PBS injected animals. Upon tumor growth, GFAP activity increased after 14 day of implantation in animals injected with tumor cells, while only background GFAP activity was observed in animals without tumor. N = 8-9 for each group, Wilcoxon rank sum test, two-sided, * P < 0.05 at day 3,7, and 28, error bar represents standard deviation.

Metastatic brain tumors also induce reactive astrogliosis in the brain [21,22], therefore, we tested whether cancer cells of non-CNS origin activate GFAP in our model. Following stereotactic injection of MDA-MB231 human breast cancer cells, we observed similar results (i.e. biphasic activation of luciferase upon tumor implantation)(Figure 3). These data indicate that the GFAP-luc; Rag2^-/- ^mice are useful tools to monitor reactive tumor-induced astrogliosis of the host compartment non-invasively.

### Real time monitoring of spatial distribution of host-mediated astrogliosis

**Figure 4:**
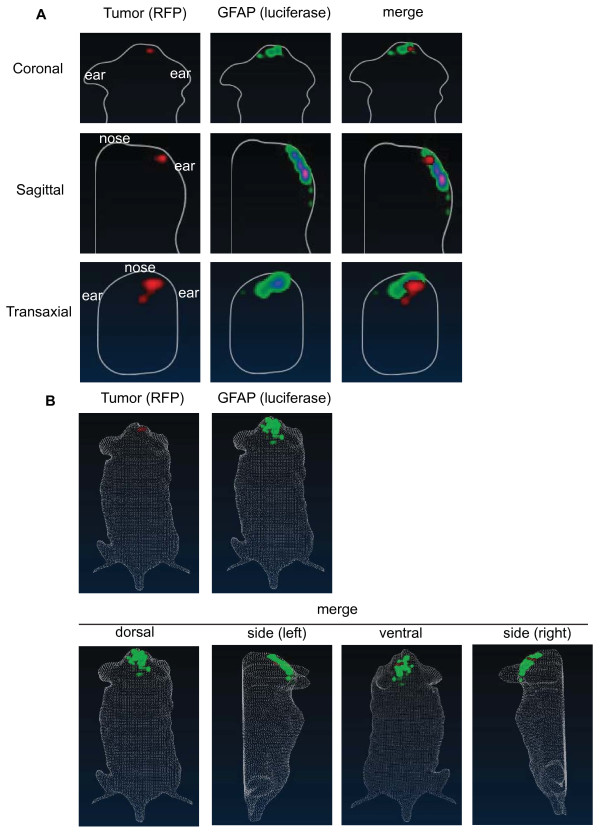
**Spatial distribution of host-mediated astrogliosis**. (A) 3D images using diffuse tomographic reconstruction algorithms for fluorescence (RFP-labeled tumor) or bioluminescent sources (GFAP activity, luciferase) were created with tumor-bearing GFAP-luc mice after a four-week incubation. Representative coronal, sagittal, and transaxial images from the center of the tumor is shown. Note the upregulation of GFAP activity adjacent to the tumor (merged image). (N = 3). (B) 3D reconstruction of fluorescent or bioluminescent sources in the whole animal is shown. While fluorescent signal from tumor was localized, GFAP activity was upregulated at the tumor periphery. Merged images from dorsal, left side, ventral and right side is shown. A representative image of three independent experiments is shown.

To understand the spatial distribution of GFAP-activation relative to the tumor in real-time, we generated a three-dimensional reconstruction of astrogliosis in tumor-bearing mice using Living Image 4.0 (Caliper Life Science). GFAP-luc; Rag2^-/- ^mice were injected with RFP-labeled DBTRG glioma cells and incubated for four weeks. At four week, the bioluminescence signals and fluorescent signals were collected and analyzed. We observed a localized tumor mass in the cortex, while GFAP activity was upregulated more broadly in the areas surrounding the tumor (Figure 4). In agreement with the immunohistochemical data (Figure 1), there was not a complete overlap between the tumor area and GFAP activity suggesting that the GFAP response in the luciferase reporter extends beyond the immediate tumor margin (Figure 4 and Additional file 1).

### GFAP activation correlates with tumor size

**Figure 5:**
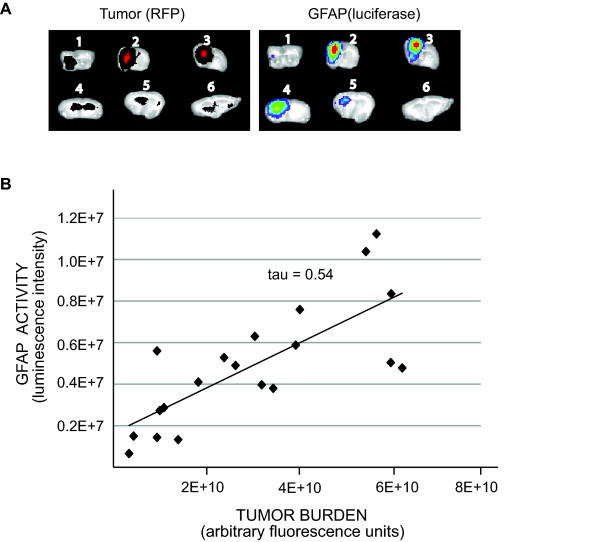
**GFAP activation correlates with tumor location and tumor size**. (A) GFAP-luc mice were injected with RFP-labeled human glioma cells, incubated for 4 weeks and six 1 mm coronal brain sections (numbered 1-6, anterior to posterior) were made for each animal. Tumor (red, left panel) and tumor-induced GFAP activation (luciferase, right panel) were localized in the same sections. A representative image of brain sections is shown. (B) Tumor burden (measured by its fluorescent intensity) and GFAP activation (measured by luciferase activity) from four tumor-bearing animals (at least five sections from each animal) were quantified. GFAP activity correlates with tumor size. Kendall's rank correlation test, two-sided, coefficient index τ = 0.54, P < 0.01.

To determine whether astrogliosis was restricted to the tumor region or a global astrocyte response was generated as a consequence of tumor growth, we monitored luciferase activity in brain sections. Following a four-week incubation of glioma in vivo, the brain was harvested and 1 mm brain sections were prepared. Consistent with the immunohistochemical data (Figure 1), reactive astrogliosis was observed only in tumor-bearing sections and sections adjacent to tumors (Figure 5A). Furthermore, we observed a statistically significant correlation between tumor size (i.e. fluorescent intensity of RFP-labeled glioma cells) and astrogliosis (i.e. GFAP-mediated luciferase activity) (Figure 5B, p < 0.01).

GFAP immunostaining (Figure 1) and our bioluminescent reporter model (Figure 4 and 5) both indicated that tumor induced-GFAP activity was mainly observed in the microenvironment of the tumor. On the other hand, GFAP activity in the contralateral hemisphere (adjacent to tumor-bearing hemisphere) was very low and close to the baseline activity of GFAP observed in control brain with no injury. Although we have found a significant correlation between the tumor size and astrogliosis in the tumor microenvironment, we did not observe a significant change of global GFAP activity (i.e. non-tumor bearing brain slabs of tumor-bearing animals) based on tumor size.

In mouse EAE and prion infection models there is a direct correlation between the amount of GFAP-mediated luciferase activity and the intensity of the insult [6,8]. Yet, in the mouse model of ischemic injury in the CNS, a correlation between the GFAP activity and infarct size was observed only in the male mice [7]. Although not a focus of these studies, we did not observe any differences in the GFAP activity upon tumor growth between male and female animals. However, other tools to monitor astrogliosis maybe useful to assess gender differences in future studies.

We established that transgenic GFAP-luc reporter mice are useful to study the interaction of the tumor growth and astrogliosis. The non-invasive nature of this model can be used for the dynamic assessment of astrogliosis during the course of tumor treatment after the delivery of chemotherapeutic drugs or early detection of a recurring tumor. Transgenic reporter models such as GFAP-luc provide insights into the molecular physiology of the tumor microenvironment which maybe useful for drug discovery or screening [23] and evaluating other transgenic models for host compartment specific response to glioma growth and invasion.

## Conclusions

Together, these results indicate that GFAP activity can be used as a surrogate marker for tumor-induced astrogliosis. Unlike the traditional methods to monitor astrogliosis, these reporter mice can be utilized for rapid, quantitative, and dynamic assessments of the tumor-host interaction.

## Methods

### Animals

GFAP-luc transgenic mice (FVB/N-Tg(Gfap-luc)-Xen) expressing the firefly luciferase gene under the control of 12 kb murine GFAP promoter were obtained from Caliper Life Sciences (Hopkinton, MA). These animals were backcrossed into a Rag2^-/- ^immunodeficient background (i.e. T and B cell deficient) for at least five generations to avoid rejections of the xenograft [9]. Mice were genotyped by quantitative PCR using primers specific for the luciferase transgene (Transnetyx Inc). Mice that did not show baseline GFAP activity (measured by luciferase activity before intracranial injection) were excluded from the study. All animal husbandry and handling procedures were approved by the University of California San Diego Institutional Animal Care and Use Committee.

### Tumor cells

Early passages of patient-derived human glioma cells, DBTRG (a kind gift from Dr. C. Kruse)[18] were used in xenograft studies. These DBTRG cells were transduced with lentivirus expressing red fluorescent protein (DBTRG-RFP) or Zsgreen (DBTRG-Zsgreen) as described earlier [10,12] to enable their identification from normal CNS parenchyma. MDA-MB-231 breast cancer cells were obtained from ATCC. All cells were maintained in Dulbecco's modified Eagle's minimum essential medium supplemented with 10% fetal bovine serum, penicillin, streptomycin, nonessential amino acids, and glutamine in a humidified atmosphere containing 5% CO_2 _at 37°C.

### Intracranial stereotactic injections

10 week-old mice were immobilized in a rodent stereotactic frame, an incision made in the skin, and a burr hole made in the skull. One million tumor cells resuspended in 5 μl of PBS were injected at a rate of 1-2 μl/minute using a Hamilton microsyringe (Hamilton, Reno, NV) mounted on a stereotactic frame (Kopf Instruments, Tujunga, CA) using coordinates of 1 mm lateral and 2 mm posterior to the bregma and 2 mm below the dura. The incision was closed with sterile sutures. Equal volume of PBS (5 μl) was used as controls.

### Quantification of bioluminescent imaging in vivo

Astrogliosis was monitored before (i.e. pre-operation) and following of incubation of 3, 7, 14, 21 and 28 days post-injection for tumor growth. Fur was removed from mice with electric clippers and Nair (Church & Dwight Co., Inc., Princeton, NJ) before imaging at each time point. Bioluminescent signals were assessed 10 minutes after interperitoneal D-luciferin injection (150 μl of 15 mg/ml stock) using a cooled charge-coupled device (CCD) camera (Spectrum; Caliper Life Sciences, Hopkinton, MA) capable of in vivo imaging (using settings of exposure time 2-10 sec, large binning, F/Stop = 1). GFAP activity was monitored by quantification of light emission from a region of interest (ROI) at each time point (Unit = radiance). Bioluminescent signal from the ear represents basal level of GFAP activity and were excluded from the ROI. Images were analyzed using Living Image software version 4.0 (Caliper Life Sciences, Hopkinton, MA).

### 3D reconstruction of bioluminescent and fluorescent signals

Gray-scale photographs and structured-light images were collected to generate a 3D reconstruction of the surface of the mice. 3D images were created using diffuse tomographic reconstruction algorithms for fluorescence (Fluorescent Imaging Tomography, FLIT) or bioluminescent sources (Diffused Luminescent Imaging Tomography, DLIT) with Living Image software version 4.0. Bioluminescent signals were assessed 10 minutes after D-luciferin injection (at the steady-state of luciferin kinetic profile).

### Immunohistochemistry

Three weeks after implantation, the animals were perfused with heparin/saline by intracardiac injection, the brains harvested and cryoembedded in O.C.T. medium (Miles Inc, Kankakee, IL). Standard immunohistochemistry was performed on cryosections (10 μm) of tumor samples using the mouse monoclonal anti-GFAP (C9205, Sigma, St.Louis, MO, 1:200) and Alexa-fluor-conjugated secondary antibodies (Invitrogen, Carlsbad, CA, 1:200). All sections were counterstained with 0.5 μg/ml DAPI (Sigma, St.Louis, MO) and tumor area defined either by the typical dense nuclei staining of the tumor (Figure 1A) or fluorescent activity of the tumor cells (Figure 1B). Immunostaining of tissue sections were imaged with an Olympus Fluoview 1000 (ASW 1.7 b) laser scanning confocal microscope equipped with 2x/0.08 N.A., 10x/0.4N.A. and 20x/0.7N.A. dry-objective lenses on a BX61 microscope (Olympus, Center Valley, PA). GFAP immunoreactivity was quantified by defining a region of interest (ROI) and by measuring the total fluorescent units in the ROI in exposure-matched images using Olympus Fluoview (ASW1.7 b) software.

### Ex vivo analyses

Four weeks after tumor implantation, tumor-bearing brain was harvested and 1 mm thick brain sections were made. Tumor burden (measured by fluorescence of RFP-labeled tumor cells) and astrogliosis (measured by bioluminescence of GFAP-luc reporter) was quantitated with a deep-cooled CCD imaging system equipped with appropriate fluorescence filter cubes with background subtraction. Images were analyzed using Living Image software version 3.1.

### Statistical analyses

All statistical analyses were performed using Mstat software (version 5.10; N. Drinkwater, McArdle Laboratory for Cancer Research, School of Medicine and Public Health, University of Wisconsin, which is available for downloading at http://www.mcardle.wisc.edu/mstat/).

## List of abbreviations

BBB: blood-brain barrier; CCD: charge-coupled device; CNS: central nervous system; EAE: experimental autoimmune encephalomyelitis; GFAP: glial fibrillary acidic protein.

## Competing interests

The authors declare that they have no competing interests.

## Authors' contributions

JL, AB and BPE designed research; JL and AKB performed research; JL and BPE analyzed data; JL, AB, and BPE wrote the manuscript. All authors read and approved the final manuscript.

## Supplementary Material

Additional file 1 **Spatial distribution of host-mediated astrogliosis**. A movie of 3D reconstruction of fluorescent (RFP-labeled tumor) and bioluminescent sources (GFAP activity, luciferase) in tumor-bearing GFAP-luc mice after a four-week incubation is shown. Click here for file
